# Controlled out-of-season spawning of reef-forming corals using offset environmental cues

**DOI:** 10.1098/rspb.2025.1558

**Published:** 2025-09-17

**Authors:** Lonidas Koukoumaftsis, Matthew Salmon, Glenn Everson, David J. Hughes, Deepa Varkey, Andrew Heyward, Andrea Severati, Craig Humphrey

**Affiliations:** ^1^ The National Sea Simulator, Australian Institute of Marine Science, Townsville, 4810 Queensland, Australia; ^2^ Australian Institute of Marine Science, Townsville, 4810 Queensland, Australia; ^3^ The University of Western Australia, Perth, 6009 Western Australia, Australia

**Keywords:** mass spawning, coral reproduction, off-season spawning, Great Barrier Reef, coral aquaculture, aquarium husbandry, out-of-season spawning, coral spawning, coral reef restoration

## Abstract

The global climate crisis has heightened the urgency for developing interventions to enhance resilience and recovery of coral reef ecosystems. However, research programmes are often bottlenecked by availability of coral early life stage material owing to the annual nature of coral mass spawning. We present a proof-of-concept of ‘out-of-season’ spawning, utilizing aquarium control technology to induce spawning in multiple Great Barrier Reef (GBR) corals held in long-term indoor aquaria. By applying a six-month offset environmental profile encompassing seasonal temperature, photoperiod and lunar cues, we induced synchronized coral spawning during austral autumn/winter between 2022 and 2023. We also ‘phase-shifted’ the hour of sunset by four hours on spawning nights, creating a more favourable time window (i.e. minimizing late nights) for gamete fertilization. Spawning occurred on comparable nights after full moon (NAFM) and at similar times after sunset (TAS) to wild conspecifics, with 2023 cohorts showing the closer alignment. Gamete fertilization was successful for six species: *Acropora millepora*, *Acropora loripes*, *Acropora hyacinthus*, *Acropora elseyi*, *Acropora austera*, and *Montipora aequituberculata*, producing *ca* 2 million larvae. We outline physiological insights into environmental regulation of coral spawning synchronicity and discuss the potential for out-of-season spawning to accelerate coral research and enhance reef restoration programmes.

## 1. Introduction

Coral reefs are hotspots of biodiversity with immense socio-economic value yet face increasing pressures globally owing to climate change [1–3] and localized impacts from coastal urbanization [4]. According to the Intergovernmental Panel on Climate Change (IPCC), up to 99% of reefs may be degraded by 2100 even under optimistic climate scenarios [5]. The coral reef crisis has intensified efforts to document acute and chronic disturbances, understand the ecophysiological responses of corals to environmental change and develop interventions to support reef resilience and recovery [6–8]. However, within populations our understanding of the physiological responses of coral larvae and recruits remains less well developed than for adult counterparts (e.g. [9]). This represents a major knowledge gap since the success of coral early life stages is critical in underpinning the long-term viability of reef assemblages at local scales [10].

A major bottleneck in improving our understanding of reef resilience and recovery is the limited opportunity to conduct coral spawning research owing to the ephemeral nature of these events. Most reef-building corals (order: Scleractinia) are broadcast spawners, exhibiting synchronous gamete release within a population often involving multiple species spawning on the same few nights each year, commonly referred to as ‘mass spawning’ events [11]. Synchronization of spawning events is a critical reproductive strategy to maximize cross-fertilization opportunities between coral conspecifics and is thought to align with optimal environmental conditions for survival, dispersal and settlement of coral larvae [12–14].

Synchronous gamete release in corals appears to be finely regulated by a sequence of environmental cues operating across hourly to seasonal timescales [15]. For example, the month in which spawning occurs is most closely correlated to seasonal variability in sea surface temperature (SST), and the specific day of spawning appears strongly linked to factors associated with the lunar cycle (e.g. tides and moonlight intensity), while the time-of-day is likely associated with the period of darkness immediately following sunset [14–18]. Regional variation in environmental cue dominance has also been observed; for example, solar rhythms appear more influential than SST in regulating spawning synchrony in Caribbean corals, indicating that the primary environmental drivers may vary by location, species or both [16].

Four decades on from the realization that most coral species are broadcast spawners [17,18], access to spawning events underpins a broad range of coral research. Spawning events are generally annual and usefully predictable—though timing can vary slightly between years at a given location [11]—however, the episodic nature of coral spawning presents challenges for scientists studying coral reproduction and recruitment. While the timing of mass coral spawning events is well documented on reefs in both hemispheres, the seasonality that is a feature of broadcast spawning at all latitudes, including equatorial reefs (e.g. [19]), limits researchers’ access to coral gametes. For species that spawn only once per year, this may reduce opportunities to a single night annually. As a result, seasonal constraints on gamete availability create a significant bottleneck for research reliant on coral early life-history stages [20], particularly when working with local species or genotypes (e.g. [21,22]).

Control over environmental parameters has been used to manipulate reproductive cycles of various marine organisms, particularly within aquaculture hatcheries [23]. Advances in aquarium control technology combined with a better understanding of the environmental regulation of coral spawning synchronicity have enabled both researchers and hobbyists alike to reliably induce spawning in aquarium corals [24]. Several Indo-Pacific coral species have now been spawned in aquaria under such conditions, typically releasing gametes on the same calendar night as their wild counterparts—often within minutes of natural spawning times [11,25].

The ability to replicate and control seasonal variability in temperature and photoperiod (i.e. photothermal manipulation [26]) enables the development of protocols to induce ‘out-of-season’ (or ‘off-season’) coral spawning. This approach could provide a reliable source of gametes and larvae at predetermined times, offering a valuable boost for coral research, yet it remains largely underexplored. We conducted an 8-year research programme culminating in the development and application of seasonally offset environmental profiles to trigger synchronized and predictable spawning of multiple Great Barrier Reef (GBR) coral species in aquaria during austral autumn/winter (May–June). This breakthrough expands larval availability beyond natural spawning windows, addressing a key limitation in coral reproduction research. We highlight how out-of-season spawning (OSS) could provide an immediate boost to coral research programmes reliant upon early life stages and opens new opportunities to investigate coral reproductive physiology.

## 2. Material and methods

### (a) Generation of broodstock

**Figure 1.**
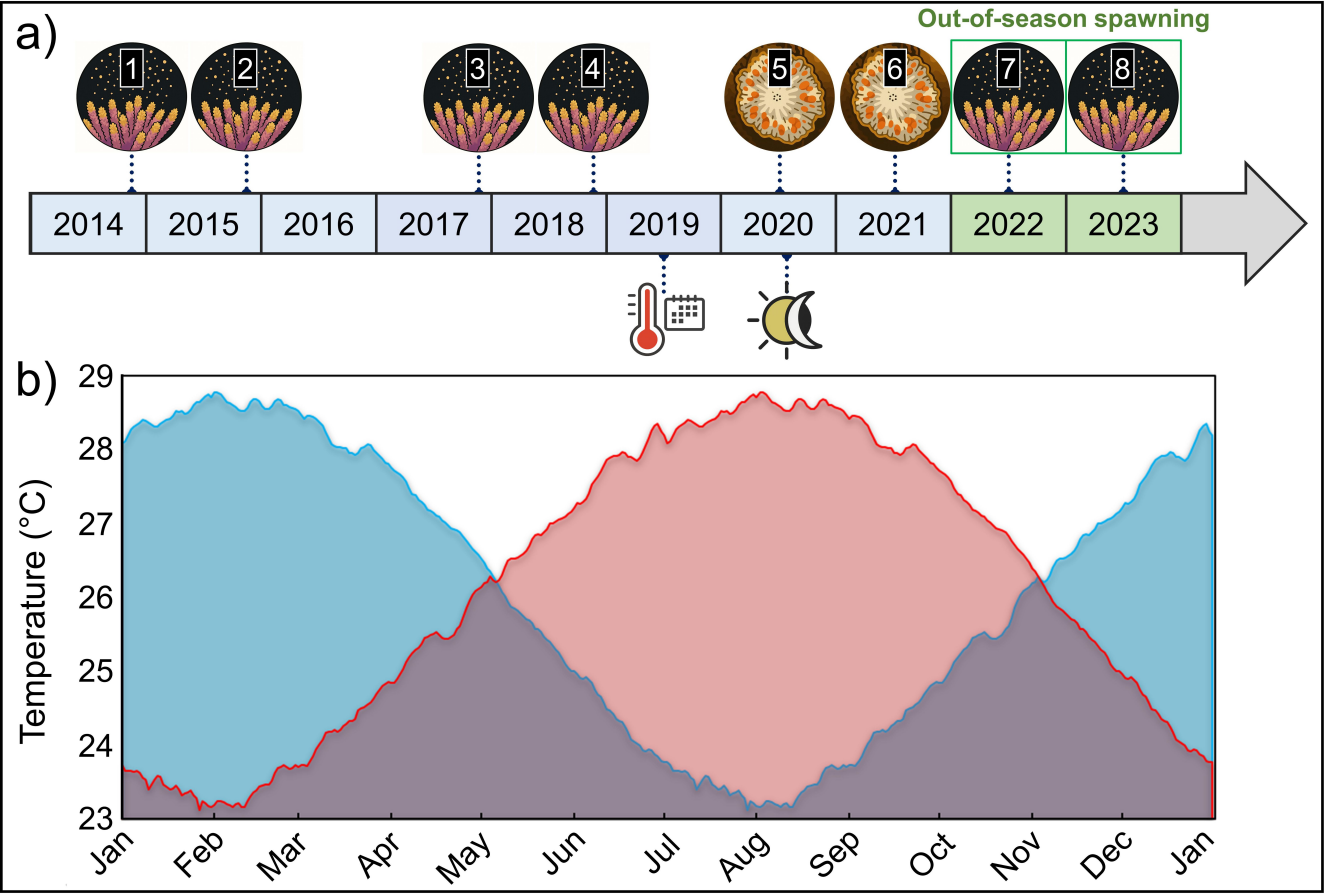
(a) Timeline of key events in the out-of-season spawning project conducted at SeaSim between 2014 and 2023. Generation of filial 1 (F1) broodstock **[1]**, *Acropora hyacinthus*; **[2]**, *Acropora loripes* and *Acropora millepora*; **[3]**, *Acropora elseyi*; **[4],** *Acropora austera* and *Montipora aequituberculata*; **[5]** and **[6]**, F1 colonies developed eggs but no gamete release observed; **[7]**, F1 coral spawning (*n* = 43), six species, 150 000 larvae; **[8],** F1 coral spawning (*n* = 59), six species, 1.7 million larvae. (b) Temperature profile for Davies Reef (central Great Barrier Reef, Australia) comprising data averaged between 1998 and 2015 (blue line) and with the application of a six-month temporal offset to generate the out-of-season temperature profile (red line). Temperature data were sourced from the Australian Institute of Marine Science (AIMS) Davies Reef weather station and correspond to 4 m depth (https://weather.aims.gov.au/#/station/4).

Experimental work was conducted at the National Sea Simulator (SeaSim), Australian Institute of Marine Science (AIMS), Townsville. First filial generation (F1) coral colonies, maintained in captivity for *ca* 5 years, were selected as broodstock owing to their demonstrated growth and survival at SeaSim. Six species were included: *Acropora hyacinthus*, *Acropora millepora*, *Acropora loripes*, *Acropora elseyi*, *Acropora austera* and *Montipora aequituberculata*. F1 corals were established from gametes obtained from parental (F0) corals collected from the central GBR at Davies Reef (mid-shelf; 18.820° S, 147.645° E), Backnumbers Reef (mid-shelf; 18.508° S, 147.148° E) and the Palm Islands region (inshore; 18.772° S, 146.532° E) between 2014 and 2018, 3−7 nights before the October, November or December full moons (Great Barrier Reef Marine Park Authority (GBRMPA) permit G21/38062.1). Typically, 12 colonies per species were collected by SCUBA divers using hammer and chisel, transported to SeaSim and held in 1200 l outdoor aquaria [27]. During annual spawning periods between 2014 and 2018 (figure 1), corals were monitored for egg/sperm bundle formation (‘setting’ *sensu* [17]) after sunset. Upon setting, colonies were isolated, and egg/sperm bundles were collected and fertilized as described in [28].

Once fertilization was verified, embryos were skimmed from the water surface, washed and transferred to 85 l culture tanks following established handling procedures [27]. Approximately one-week post-fertilization, larvae were settled (see electronic supplementary material, S1.1) onto 20 mm aragonite plugs (Ocean Wonders) that had been pre-conditioned for eight weeks in outdoor aquaria to achieve 30–60% coverage of crustose coralline algae—a known cue that induces the coral settling response [28–30]. Three days after settlement, polyvinyl chloride (PVC) plug trays (electronic supplementary material, figure S1) containing settled recruits were transferred to indoor community, semi-closed aquaria (280 l, provided with three system turnovers of seawater per day). These community aquaria were equipped with a protein skimmer to remove organic waste and augment gas exchange [31], while mechanical filtration was provided by a media filter system containing glass beads (0.7 µm diameter). To facilitate uptake of photosynthetic endosymbionts (family: Symbiodiniaceae), fragments of F0 parental colonies were introduced as donor colonies alongside newly settled recruits [32]. Corals were maintained in community aquaria under a constant temperature regime (27.3 ± 0.3°C) for 5 years (2014−2019) before being moved to a dedicated experimental holding room.

### (b) Experimental set-up—out-of-season spawning

**Figure 2.**
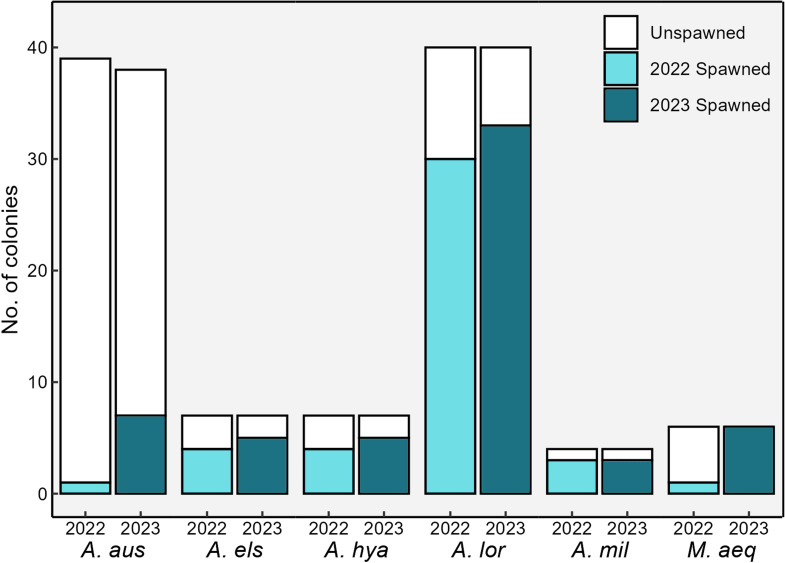
Numbers of F1 broodstock colonies per species and proportion of colonies that spawned during the 2022 (light teal) and 2023 (dark teal) out-of-season spawning events. Note: a slight decrease in *Acropora austera* colonies occurred between 2022 (*n* = 39) and 2023 (*n* = 38) owing to mortality. Species abbreviations*: A. aus*, *Acropora austera*; *A. els, Acropora elseyi; A. hya, Acropora hyacinthus; A. lor, Acropora loripes; A. mil, Acropora millepora; M. aeq, Montipora aequituberculata*.

Adult F1 corals (see figure 2 for numbers and species) were moved to a dedicated experimental system with the capability to dynamically manipulate (and offset) key environmental variables, including water temperature, photoperiod and moonlight. Corals were distributed across two aquarium systems, each comprising a pair of 1200 l tanks connected to a shared 700 l sump (see electronic supplementary material, S1.2 and figure S2 for system technical details). Each system was supplied with 1 µm filtered seawater and maintained at a salinity of 35 ± 1 practical salinity units (PSU - dimensionless). Seawater was continuously exchanged at a rate of 1.7 system turnovers per day to ensure stability of key water chemistry parameters essential to coral growth (e.g. alkalinity, calcium and magnesium, see [31,33]) and minimize nutrient loading (see electronic supplementary material, figure S3).

Corals were provided with a range of daily feeds, including microalgae (comprising a mixture of *Dunaliella* sp., *Tisochrysis lutea* and *Chaetoceros muelleri*) at a final density of 2000 cells ml^−1^, HUFA-enriched instar II *Artemia salina* (0.5 nauplii ml^−1^), unenriched instar I *A. salina* (0.5 nauplii ml^−1^) and enriched rotifers (0.35 nauplii ml^−1^). Corals were provided a maximum photon irradiance (400–700 nm) of 150 µmol photons m^−2^ s^−1^ on a 12 : 12 photoperiod. One system was fitted with 16 commercial LED panels (Hydra^®^ 52, Aqua Illumination^®^, Ames, IA, USA), and the other with eight custom LED panels (see electronic supplementary material, figure S2 for respective spectra). Temperature was held at 27.3°C to match previous holding conditions until the OSS temperature profile was applied (figure 1).

### (c) Applying an ‘out-of-season’ offset temperature profile

To create a representative seasonal temperature profile, historical temperature data were sourced from the AIMS weather station at Davies Reef (18.832° S, 147.635° E). Specifically, data from 4 m depth between 1998 and 2015 were averaged to create a composite seasonal temperature profile representative of the central GBR region (figure 1; electronic supplementary material, data S1). This composite profile was then uploaded as a lookup file to a programmable logic controller (PLC) permitting dynamic control of aquarium water temperature (electronic supplementary material, figure S4). To achieve a seasonal offset, we advanced the PLC system date by six months, so daily lookup temperature values were six months ahead of the local date. On 4 June 2019 (when temperatures were identical in both the offset and standard profiles), the out-of-season temperature profile was initiated (figure 1; electronic supplementary material, figure S4). Out-of-season temperature profiling ran for 13 months from June 2019 to July 2020, after which seasonally offset profiling of photoperiod and moonlight was incorporated (figure 1).

### (d) Offsetting photoperiod and moonlight

In July 2020, a six-month offset photoperiod profile was initiated, corresponding to the six-month future photoperiod at Townsville, Australia (https://www.timeanddate.com). A description of photoperiod terminology is provided in electronic supplementary material, S1.3. We also phase-shifted the photoperiod, by advancing the PLC lookup time to bring first light, sunrise, sunset and last light forward by 4 h (see electronic supplementary material, figure S5) to provide a more convenient time window for researchers to observe spawning and harvest gametes. To reproduce the low natural levels of moonlight on reefs [34], we used a custom white LED strip with a diffuser facing the ceiling to provide scattered incident light. Moonlight intensity was calibrated to a maximum of 1 lx at full moon, verified using a portable lux sensor (3281A, Yokogawa, Tokyo, Japan). Moonlight profiling was achieved using a dimmable LED driver, delivering a percentage of 1 lx relative to the lunar phase. A moonlight profile was generated from six-month future moonlight data (https://www.timeanddate.com) and loaded into the PLC to control moonrise and moonset times with a 4 h phase shift aligned to the solar photoperiod (electronic supplementary material, figure S6). Importantly, because corals were held under a fixed lighting regime over multiple years, we adjusted photoperiod to reflect seasonal changes in daylength but kept peak daily irradiance constant year-round. Environmental profiles (photoperiod, lunar period and temperature) are available as CSV files in the electronic supplementary material.

### (e) Verifying gametogenesis, F1 spawning and F2 generation

**Figure 3.**
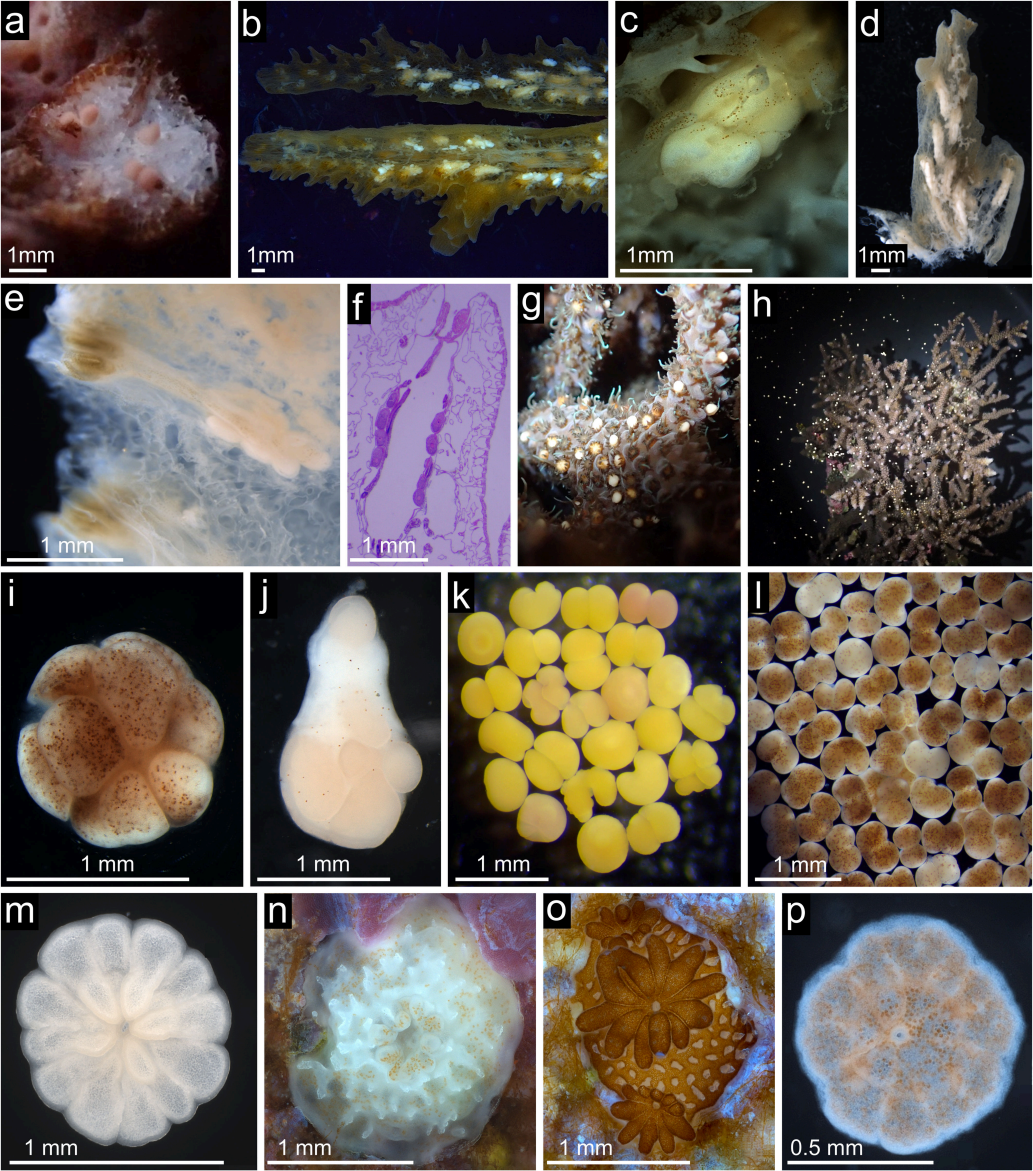
Representative images of coral early life stages and spawning events in the out-of-season coral spawning programme. (a) Pigmented eggs in *Acropora hyacinthus* (June 2020). (b–e) Decalcified samples showing gametogenic development in: (b) *Acropora millepora* (June 2020), (c) *A. hyacinthus* (June 2020), (d) *A. hyacinthus* (May 2021), (e) *Acropora loripes* (May 2021). (f) Histological section of *A. loripes* showing developing eggs (April 2022). (g) *A. millepora* adult colony ‘setting’ (May 2022). (h) Bundle release from *A. millepora* (May 2022). (i) *Montipora aequituberculata* gamete bundle (June 2022). (j) Dissociation of *A. loripes* gamete bundle (June 2022). (k,l) Fertilized embryos of *A. millepora* (May 2022) and *M. aequituberculata* (May 2023). (m–p) Second filial generation coral recruits of: (m) *A. millepora*, (n) *A. hyacinthus*, (o) *A. loripes* (19 days post-settlement, with Symbiodiniaceae visible) and (p) *M. aequituberculata*.

In the week prior to expected spawning in May (2020−2023), two 3−4 cm nubbins per colony were sampled from selected *A. loripes, A. hyacinthus, A. elseyi*, *A. millepora, A. austera and M. aequituberculata* colonies. Nubbins were fixed in a 4% formaldehyde–saltwater solution for 24 h and then decalcified in 3% HCl–saltwater solution. Samples were subsequently cross-sectioned, and oocyte development was examined by microscopy (figure 3).

During the 2022 and 2023 spawning events, F1 broodstock corals were observed and isolated where ‘setting’ was apparent; gametes were collected and fertilization was attempted and assessed. Fertilization success was determined as the proportion (%) of embryos relative to the total egg sample. Larval cultures were created, and a subset of second filial generation (F2) larvae were settled to attempt life cycle closure (see electronic supplementary material, S1.4).

### (f) Comparative assessment of gamete and larval physiology

To evaluate whether gametes and larvae produced under OSS conditions were physiologically comparable to those from natural mass spawning events, we compared several key larval performance traits—fertilization success, settlement rates and oocyte diameter—against published values for conspecifics. This comparison was performed using a targeted meta-analysis of available studies reporting coral reproductive metrics for the six GBR species used here. See electronic supplementary material, S1.5–S1.8 for full details of search terms, filtering criteria and methodology.

### (g) Statistical analyses

Statistical analyses were performed in R v. 4.4.0 using the stats, emmeans and tidyverse packages. Analyses aimed to assess variation in coral spawning timing across years and species, and to evaluate alignment between OSS and natural benchmarks. Spawning timing was quantified using two biologically meaningful metrics: nights after full moon (NAFM) and time after sunset (TAS), analysed as raw values to retain biological interpretability. To satisfy the distributional assumptions of Poisson models (i.e. non-negative integer response), a constant of 1 was added to all NAFM values. For TAS models, the response variable was defined as TAS in minutes (rounded to the nearest whole minute) to meet integer requirements of the Poisson distribution. A categorical predictor variable (‘dataset’) was defined with three levels: 2022, 2023 and a reference dataset compiled from wild spawning observations [11]. Generalized linear models assuming a Poisson distribution and log link function were fitted separately for NAFM and TAS in each species, using 'dataset' as the model term. Where significant effects remained after Bonferroni correction for multiple testing, pairwise comparisons of estimated marginal means were performed with an additional Bonferroni correction.

## 3. Results

### (a) Spawning observations (by year)

#### (i) 2020–2021

No coral spawning was observed during 2020 or 2021. However, in June 2020, histological and visual evidence confirmed late-stage gametogenesis in one *A. hyacinthus* and one *A. millepora* colony (figure 3a–c), suggesting that spawning may have occurred unobserved. The absence of apparent stressors makes gamete reabsorption appear less likely [35,36]. In 2021, developing eggs were again present in two *A. hyacinthus* and five *A. loripes* colonies (figure 3d,e), but no spawning was detected. Subsequent investigation revealed a moonlight cue delivery error that stemmed from a misalignment in the lookup process, causing the control system to retrieve moonrise/moonset from incorrect dates despite a correct underlying dataset. As a result, moonrise/moonset times were incorrect for any given day in the three months leading up to spawning, when, critically, the full moon (1 lx intensity) cue was not delivered on the correct date.

#### (ii) 2022

A total of 43 out of 109 coral colonies successfully spawned in the OSS programme during 2022 (figure 2). Spawning was split across two months (May/June), with the majority (38 of 43) of colonies spawning in June. Between 13 and 16 May (6−9 NAFM) four coral species spawned: three colonies of *A. elseyi*, two each of *A. millepora* and *A. loripes*, and a single *A. austera* colony (table S1). Some colonies exhibited partial spawning across successive nights (e.g. one *A. millepora* colony spawned over three consecutive nights) (electronic supplementary material, table S1). Fertilization success ranged from 32% (*A. elseyi*) to 80% (*A. millepora*) (table 1; electronic supplementary material, table S2). Fertilization was not attempted for *A. loripes* owing to limited bundle release during partial spawns, or for *A. austera,* where only one colony spawned. In total, approximately 25 000 larvae were produced in May: 15 000 from *A. millepora* and 10 000 from *A. elseyi* (table 1).

**Table 1.** Larval production from out-of-season spawning events (2022−2023), showing the number of larval cultures established, fertilization success (% range), and total number of larvae produced per species and month. Superscript numbers denote the number of genotypes (i.e. distinct F1 colonies) contributing to each data point. 'Total larvae' includes all cultures, including those for which fertilization success was not determined (n.d.). Species abbreviations as per figure 2.

	May			June			
	no. cultures	fert. range (%)	total larvae	no. cultures	fert. range (%)		total larvae
**2022**							
*A. aus*	—	—	—	—	—		—
*A. els*	1^[3]^	32^[3]^	1.0 × 10^4^	1^[3]^	n.d.		1.3 × 10^4^
*A. hya*	—	—	—	1^[3]^	n.d.		1.5 ×10^4^
*A. lor*	—	—	—	5^[3–13]^	65^[3]^–77^[9]^		8.2 × 10^4^
*A. mil*	1^[2]^	80^[2]^	1.5 × 10^4^	1^[2]^	n.d.		1.5 × 10^4^
*M. aeq*	—	—	—	—	—		—
**2023**							
*A. aus*	1^[3]^	n.d.	2.5 × 10^4^	—	—		—
*A. els*	1^[2]^	n.d.	5.0 × 10^4^	1^[3]^	76^[3]^		6.6 × 10^4^
*A. hya*	—	—	—	1^[3]^	83^[3]^		3.1 × 10^4^
*A. lor*	—	—	—	3^[9–17]^	84^[9]^–92^[17]^		1.5 × 10^5^
*A. mil*	1^[2]^	n.d.	2.5 × 10^4^	2^[2]^	78^[2]^		2.3 × 10^4^
*M. aeq*	2^[3–6]^	11^[3]^–91^[6]^	1.2 × 10^6^	2^[3–5]^	94^[3]^		2.1 × 10^5^

The second spawning event occurred between 10 and 17 June 2022 (4−11 NAFM), and involved five species: 28 colonies of *A. loripes*, four *A. hyacinthus*, three *A. elseyi*, two *A. millepora* and one *M. aequituberculata* (figure 3i,j). As in May, several colonies exhibited partial spawning over multiple nights (electronic supplementary material, table S1). Five larval cultures were established for *A. loripes* across five nights (4−9 NAFM) with gamete contributions from 3−13 colonies. Single cultures were also produced for *A. elseyi*, *A. millepora* and *A. hyacinthus* using gametes from 2 or 3 colonies each. In total, *ca* 125 000 larvae were produced in June 2022 (table 1).

Overall, the highest proportion of spawning F1 broodstock was observed in *A. loripes* and *A. millepora* (75%), followed by *A. hyacinthus* and *A. elseyi* (both 57.1%), *M. aequituberculata* (16.7%) and *A. austera* (2.6%) (figure 2). Larval cultures were successfully established for four of the six species (*A. millepora, A. elseyi*, *A. loripes,* and *A. hyacinthus*), yielding approximately 150 000 larvae in total (table 1). These larvae were subsequently settled, enabling us to close the life cycle of these four species entirely within SeaSim (figure 3m–o).

#### (iii) 2023

More colonies (59 of 108) spawned in 2023 compared with the previous year (figure 2). As in 2022, spawning was split across May and June, with the majority (50 of 59) of colonies spawning in June. Between 3 and 7 May (2–6 NAFM), 16 colonies across four species spawned, with most colonies exhibiting partial spawning over multiple nights (electronic supplementary material, table S1). Fertilization was successful for all species, with the highest fertilization success observed in *M. aequituberculata* (91%; table 1 and figure 3l). Approximately 1.3 million larvae were produced during this spawning event, the majority of which (1.2 million) were from *M. aequituberculata*.

A second spawning period occurred between 31 May and 8 June 2023 (1−9 NAFM), involving 50 colonies from six species (electronic supplementary material, table S1). Partial spawning across multiple nights was again observed in some individuals. Fertilization was successful across all species, with *M. aequituberculata* again yielding the highest fertilization success (94%, table 1). Among *Acropora* species, fertilization success ranged from 76% (*A. elseyi*) to 92% (*A. loripes*) (table 1). June spawning produced approximately 475 000 larvae.

Across both spawning events, successful larval settlement was achieved for *A. austera* and *M. aequituberculata,* closing the life cycle of these species in captivity for the first time (figure 3p). The highest proportion of spawning F1 broodstock was in *M. aequituberculata* (100%) followed by *A. loripes* (83%), *A. millepora* (75%), *A. hyacinthus* and *A. elseyi* (both 71%), and *A. austera* (18%) (figure 2). Approximately 1.7 million larvae were produced across six species in 2023, representing an 11-fold increase compared with 2022 (table 1). Comparative analysis of reproductive traits showed that OSS-spawned corals exhibited fertilization success, settlement rates and egg sizes broadly consistent with or exceeding published values for wild conspecifics (see electronic supplementary material, S3.1 and tables S2−S7). These results indicate that OSS-derived larvae performed within the expected physiological range observed in natural spawning events.

### (b) Spawning synchrony

**Figure 4.**
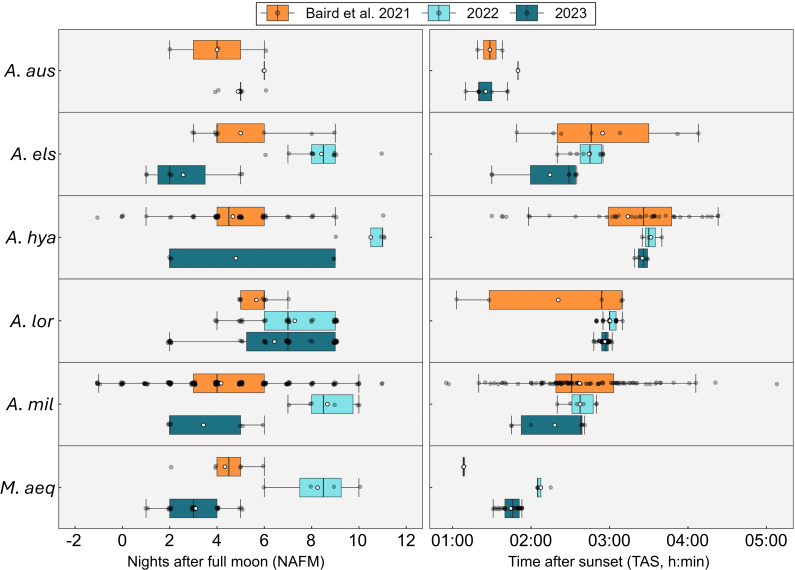
Comparison of out-of-season coral spawning with published natural spawning data from the Great Barrier Reef (GBR) (Baird *et al.* 2022 [11]). (a) Nights after full moon (NAFM) and (b) time after sunset (TAS) for each species in 2022 and 2023 compared with GBR observations from the Indo-Pacific coral spawning database [11]. Natural spawning data were filtered to match reef zones of origin (inshore or mid-shelf) for each F0 broodstock species. NAFM sample sizes: *Acropora elseyi* (*n* = 9), *Acropora hyacinthus* (*n* = 28), *Acropora loripes* (*n* = 9), *Acropora millepora* (*n* = 43), *Montipora aequituberculata* (*n* = 6), *Acropora austera* (*n* = 3). Three anomalous *A. elseyi* values (−14 NAFM) were excluded. Time after sunset sample sizes: *A. austera* (*n* = 2), *A. elseyi* (*n* = 7), *A. hyacinthus* (*n* = 20), *A. loripes* (*n* = 5), *A. millepora* (*n* = 21), *M. aequituberculata* (*n* = 2). Species abbreviations as per figure 2.

To assess how OSS aligned with natural reproductive timing, raw values of NAFM and TAS were compared across 2022, 2023 and a published wild dataset [11]. For NAFM, spawning in 2022 occurred significantly later than in 2023 for two of the six species (*A. hyacinthus* and *A. millepora*). Compared with natural values reported by Baird *et al.* [11], NAFM in 2022 was also later for *A. hyacinthus* and *A. millepora*. In contrast, there were no significant differences in NAFM detected between 2023 and the Baird *et al.* [11] dataset, indicating that the 2023 cohort showed close alignment with natural spawning windows (figure 4).

For TAS, 2022 spawning occurred significantly later in the evening in 2022 than in 2023 for *A. elseyi* and *M. aequituberculata* (electronic supplementary material, table S9). When compared with Baird *et al.* [11], TAS was significantly greater (i.e. spawning occurred later in the evening) in both 2022 and 2023 for *A. loripes* and *M. aequituberculata*, suggesting a persistent shift in diel timing (figure 4). However, in *A. elseyi* and *A. millepora*, TAS in 2023 was significantly earlier than in the wild dataset, indicating a shift to earlier spawning in the evening. Together, these results suggest that temporal synchrony with natural spawning improved in 2023, particularly in relation to full moon timing (NAFM), although species-specific variation in diel timing (TAS) persisted. Full statistical outputs are provided in electronic supplementary material, tables S8–S13.

## 4. Discussion

Research on early coral life stages often requires access to coral gametes, larvae and recruits. However, since many coral species are annual broadcast spawners, availability of such material is limited to a narrow seasonal window, creating a bottleneck for research programmes. Here, we utilized advanced aquarium technology to replicate and offset the seasonality of environmental cues (temperature, photoperiod and moonlight), inducing predictable spawning of GBR corals in austral winter—successfully generating nearly 2 million coral larvae. We discuss the physiological insights gained into environmental regulation of coral spawning and how OSS programmes could accelerate research into coral early life history and processes.

### (a) Insights into environmental regulation of spawning synchronization

Corals maintained in the out-of-season environmental profile spawned six months apart from their natural reef counterparts as expected (i.e. May/June rather than November/December). Spawning timing closely matched literature observations of natural mass spawning events, particularly in 2023, with observed NAFM and TAS values aligning closely with wild conspecifics [11,18]. The close match in spawning timing supports growing evidence [25,33,37,38] that temperature, sunlight and moonlight are the primary environmental cues regulating coral mass spawning, indicating that the environmental profile effectively replicated these drivers to induce spawning synchrony. Although we did not simulate tidal patterns within our aquarium systems, spawning still occurred close to previously reported timings, suggesting that while tidal patterns may be correlative with those cues for spawning synchronicity, they may have minimal direct effect on the coral taxa examined here. However, we are unable to exclude the possibility that location-specific conditions—such as site of origin—may have contributed to this observed outcome. Further development of the system described here could allow greater manipulation of these parameters to better understand endogenous (circadian rhythms) and exogenous controls that drive spawning synchrony in corals.

Interestingly, pigmented eggs were observed in two of the larger coral colonies in June 2020, <12 months after offset temperature profiling was initiated. This supports observations that temperature change is strongly linked to onset of gametogenesis and indicates capacity for some corals to synchronize to a modified temperature profile inside a year. During the following year (2021), offset day length and moonlight profiles were incorporated into the system. Subsequently, a total of eight coral colonies from three species were observed to have pigmented eggs one week prior to expected spawning dates, again suggesting corals were synchronized to the modified (offset) environmental profile. However, no gamete release was observed for any taxa over the expected spawning period (i.e. 0–11 NAFM, June/July)—presumably owing to issues with moonlight profiling (see Material and methods). While this outcome was unfortunate for the experiment, it did appear to reinforce the importance of moonlight within the hierarchy of cues coordinating synchronous gamete release (e.g. [25,39,40]). Nightly irradiance from moonlight modulates circadian rhythms in corals [41,42] and the significance of lunar periodicity has long been recognized in coral reproductive studies. Moonlight may also be regulating synchronicity in corals at finer temporal scales. It is plausible, as found in some other marine invertebrates (e.g. [43]), that diurnal exposure to moonlight may aid in regulating the precise timing of spawning in corals. It is also important to note that we were unable to disentangle the contribution of colony age when comparing spawning observations between years, so this should also be considered when interpreting results presented here.

In contrast to the evidence suggesting a role for moonlight in regulating spawning synchronization, the importance of solar insolation in this study is less clear. While the photoperiod was dynamically profiled to match seasonal changes, maximum daily light intensities were kept constant during the experiment, with a peak intensity of 150 µmol photons m^−2^ s^−1^ (matching long-term growth conditions). As a result, corals received a lower and less seasonally variable daily light dose than would occur on natural reefs [44–46]. Thus, while it appears that full replication of seasonal insolation variance is not required to achieve predictable spawning for the coral taxa here—and thus may be relatively unimportant as an environmental cue—we acknowledge that it is not possible to tease apart any effect(s) of photoperiod modulation. Certainly, insolation is thought to serve as an important spawning cue in low-latitude reefs, where annual SST variability is inherently lower than tropical reef systems [47], and where its importance as a cue in higher-latitude reef systems such as the GBR is presumably overshadowed by that of SST variance.

**Figure 5.**
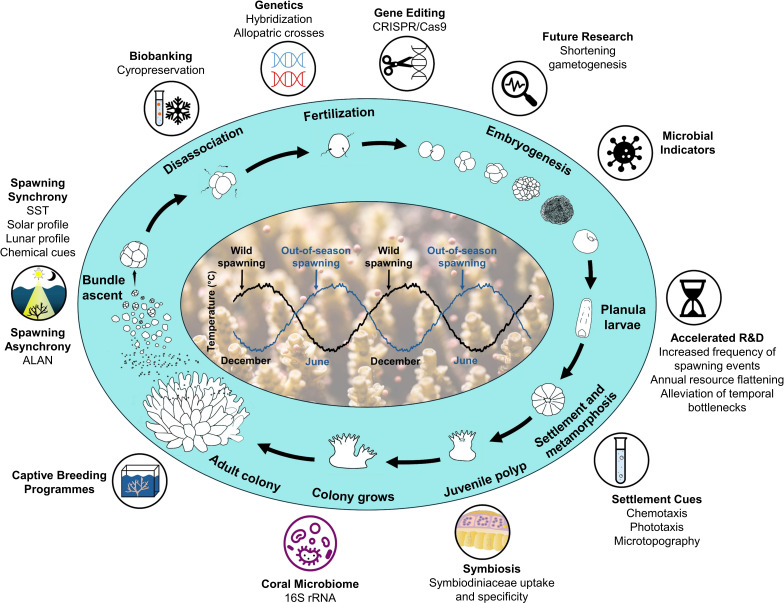
Potential research applications for out-of-season coral spawning. (a) Acceleration of current research (e.g. gene editing, biobanking, symbiont uptake, larval settlement cues discovery). (b) Novel applications such as coral hybridization opportunities and shortening of gametogenesis cycles. (c) Use of spawning systems as experimental platforms to manipulate spawning cues (e.g. chemical cues, lunar profile, solar profile, sea surface temperature (SST), artificial light at night (ALAN)). Teal shading indicates relevant coral life stages adapted from [6,48].

Out-of-season spawning may also provide a useful platform to examine environmental factors contributing to asynchronization of coral spawning (figure 5). For example, artificial light at night (ALAN) is an emerging threat to coastal reef systems, altering diel cycles of organisms including corals, modifying coral polyp feeding behaviour [49] and causing delayed gametogenesis among key broadcast spawning coral species [40,50–52]. To date, evidence of ALAN disrupting coral spawning is mostly observational, yet OSS programmes offer a clear experimental pathway to test these *ex situ*.

### (b) A tool to accelerate research into coral early life-history stages

Out-of-season spawning could accelerate research efforts (figure 5) by theoretically allowing year-round access to study coral early life stages and processes, limited primarily by logistics (space, cost and effort), rather than fundamental organismal biology *per se*. However, the suitability of *ex situ*, out-of-season coral larvae for research—especially ecophysiological studies (e.g. [53])—depends on whether physiological differences exist compared with naturally spawned larvae. Previous work has shown that various aspects of larval physiology (developmental timing, energy reserves, competency, recruitment success and growth) are influenced by environmental history and impacted by environmental stress (e.g. [54–56]). Whether synchronization to offset environmental profiles confers additional physiological stress to the coral that subsequently impacts larval quality is unknown and represents a critical area for further investigation.

To assess whether larvae produced in out-of-season conditions are physiologically comparable to those from natural mass spawning events, we compared several key metrics—including fertilization success, settlement rate and oocyte diameter—with published values from the literature (electronic supplementary material, tables S2–S7). Across five of six species, physiological traits fell within or exceeded reported ranges, suggesting health and normality under offset environmental profiles are broadly consistent with *in situ* observations. While some variation was observed (e.g. lower fertilization success in *A. elseyi*), these differences were relatively minor, offering preliminary support for the viability of OSS-derived larvae in ecophysiological and restoration applications; however, more detailed investigation of potential physiological trade-offs is warranted.

While understanding how *ex situ* offset environmental profiles influence coral larval fitness is an important future step, several applications already exist where ‘out-of-season’ coral larvae could provide an immediate research boost (figure 5). For example, CRISPR/Cas9 genome editing—applied to coral zygotes via microinjection—is highly time and labour-intensive [20], so a longer time window for zygote availability could catalyse significant advances in genetics and gene-function studies. Year-round access to coral gametes could also support other research areas, such as workflows and processes aimed at identifying biological and chemical cues for inducing larval settlement (e.g. [30]). Shortening the gametogenesis cycle through further environmental manipulation could also reduce the time between captive spawning and represents an interesting but unexplored research avenue. It would, however, be prudent to explore any associated physiological trade-offs (e.g. [57]) in parallel.

### (c) Towards an improved understanding of coral husbandry and nutrition

Here we report long-term maintenance of key GBR corals in a controlled aquarium environment, including full life-cycle closure. While species-specific requirements for captivity likely exist [58], the aquarium conditions and nutritional regimes applied here provide a strong foundation for the long-term husbandry of diverse GBR coral taxa. However, scope for improvement may exist, as evidenced by occasional observations of neutrally buoyant egg/sperm bundles in *A. loripes*—a phenomenon rarely reported in nature for this species. Positive buoyancy is a key trait ensuring optimal fertilization and dispersal of egg/sperm bundles [59–62], and wax esters—a type of lipid involved in energy storage—are the main contributor to positive buoyancy in coral eggs [60,63]. Previous examinations of tissue macromolecular composition in corals both before and after spawning further showed that most of the energy invested into coral reproduction originates from lipid metabolism [64]. Although lipid content and composition of gametes were not explicitly examined here, previous work suggests heterotrophic feeding and high light drive increased coral lipid content [65–68]. Here, we provided a selection of heterotrophic feeds (microalgae, rotifers and *Artemia salina*), spanning *ca* 15−750 µm in size to suit the variable polyp sizes across the six coral species in this experiment (*ca* 0.4−1.6 mm [69]). However, as feeding regimes for captive corals continue to evolve [70–72], we are unable to rule out nutritional deficiency as a factor in the decreased buoyancy of egg/sperm bundles observed in *A. loripes*.

In addition to heterotrophy, light has been identified as a key limiting resource governing energy investment into reproduction for reef-forming corals [73]. Specifically, Leuzinger *et al.* [73] found that partial shading (equivalent to *ca* 35% incident irradiance) of *Montipora digitata* colonies at Heron Island (southern GBR) caused decreased energy investment into reproduction compared with unshaded corals, while colonies subjected to full shading (*ca* 3% incident irradiance) halted gametogenesis altogether. Thus, energy allocation strategies in corals are inherently flexible and depend on the amount of light relative to their photoacclimation status [74]. In this study, light levels provided were sufficient to facilitate energy allocation towards reproduction for all six coral species, but whether energy allocation differences from photoacclimation status may have contributed to observed neutral buoyancy in *A. loripes* egg/sperm bundles is unknown. Likely, the total daily photon dose provided (*ca* 5.5 mol photons m^−2^ d^−1^) was lower than that available to corals *in situ* at their respective depth and site of origin (see [46]), so it is plausible that energy investment into reproduction for *A. loripes* (and other species examined) could be boosted by increased irradiance.

Notably, we observed lower spawning rates among F1 broodstock of *A. austera* compared with other *Acropora* species (figure 2). Given species-specific differences in age to maturity within Acroporidae [75], and the fact that *A. austera* colonies were the youngest in our spawning system (settled November 2018), it is possible that most individuals had not yet reached sexual maturity, which typically averages 3.9 years but can extend to *ca* 5 years in some taxa [75]. Thus, age-related factors should be considered alongside nutrition and light when interpreting the reproductive performance presented here.

### (d) Out-of-season spawning—viability as a tool to support reef restoration efforts

Global degradation of coral ecosystems has resulted in a surge in active reef restoration initiatives aimed at supplementing traditional passive habitat protection measures [76]. Outplanting of corals onto degraded reefs is a common strategy to increase coral cover and enhance reef architectural complexity [76]. While outplanting programmes often rely on asexual propagation, or ‘fragments of opportunity’ from nearby colonies, the use of sexually propagated coral recruits in reef restoration programmes can boost climate resilience through enhanced genetic diversity [77]. Therefore, coral aquaculture—i.e. raising large numbers of corals in captive aquaria—is becoming an increasingly important component of the reef restoration toolbox [77,78].

To date, a role for coral aquaculture has involved the collection of gametes, which are fertilized, settled onto artificial structures and reared *ex situ* before outplanting (e.g. [79]), or using gametes that are collected and fertilized in temporary holdings before controlled release onto designated reef areas (e.g. [80]). OSS necessitates year-round holding of broodstock in aquaria under environmental control, which may impose techno-economic constraints for alignment with outplanting efforts. Specifically, the initial cost of aquarium equipment and ongoing maintenance, energy costs to maintain temperature against ambient conditions and provision of year-round husbandry impact cost-effectiveness. However, OSS programmes allow outplanting of coral recruits at different times of the year (pending acclimation to ambient temperature), when physicochemical (light, currents, sediments) and biological (planktonic supply, corallivory) selective pressures may be more favourable, thus boosting relative return on effort (RRE) [81] via increased survivorship. Growing evidence suggests epigenetic changes arising from exposure to favourable biological and physical conditions at specific coral nurseries during early life stages enhance corals’ ability to endure environmental perturbation [82], but whether similar benefits could be derived by outplanting coral recruits out-of-season is unknown. As coral cryopreservation technologies advance rapidly [83], OSS programmes offer a platform to test outplanting viability for (reanimated) larvae outside natural spawning windows as part of possible future restoration efforts [84].

### (e) Future directions

This study marks the first successful demonstration of ‘out-of-season’ spawning for GBR corals through manipulation of environmental cues in aquaria. Our findings demonstrate that OSS can support high fertilization rates and larval settlement across multiple taxa, producing millions of viable larvae under controlled indoor conditions. The ability to generate coral gametes and larvae on demand could accelerate existing research programmes, particularly for reproductive ecology and reef interventions, while creating novel research opportunities (figure 5). For example, the ability to precisely control the time of spawning for specific coral species raises the intriguing possibility of exploring hybridization opportunities between corals that are otherwise temporally separated in their spawning window(s). This could provide an opportunity to create novel coral phenotypes (e.g. [81]), potentially with outbreeding enhancement (or hybrid vigour, see [85,86]) that could boost local reef resilience if planted out onto degraded reef areas.

Out-of-season spawning represents an underexplored technology for corals, with novel applications likely continuing to emerge over time. As such, we expect OSS programmes to form a key part of the growing toolbox of active intervention strategies aimed at reversing the global decline of coral reefs.

## Data Availability

Supporting data are available on Dryad [87]. Datasets and materials supporting this article have been uploaded as part of the supplementary material [88].

## References

[1] Hoegh-Guldberg O, Poloczanska ES, Skirving W, Dove S. 2017 Coral reef ecosystems under climate change and ocean acidification. Front. Mar. Sci. **4**, 252954. (10.3389/fmars.2017.00158)

[2] Hughes DJ, Alderdice R, Cooney C, Kühl M, Pernice M, Voolstra CR, Suggett DJ. 2020 Coral reef survival under accelerating ocean deoxygenation. Nat. Clim. Chang. **10**, 296–307. (10.1038/s41558-020-0737-9)

[3] Hughes TP *et al*. 2018 Spatial and temporal patterns of mass bleaching of corals in the Anthropocene. Science **359**, 80–83. (10.1126/science.aan8048) 29302011

[4] Rosenberg Y *et al*. 2022 Urbanization comprehensively impairs biological rhythms in coral holobionts. Glob. Chang. Biol. **28**, 3349–3364. (10.1111/gcb.16144) 35218086 PMC9311646

[5] IPCC. 2018 Summary for Policymakers. In Global warming of 1.5°C. An IPCC special report onthe impacts of global warming of 1.5°C above pre-industrial levels and related global greenhouse gasemission pathways, in the context of strengthening the global response to the threat of climate change,sustainable development, and efforts to eradicate poverty (ed. V Masson-Delmotte, *et al*). In Geneva, Switzerland: World Meteorological Organization. (10.1017/9781009157940.001)

[6] Randall C *et al*. 2020 Sexual production of corals for reef restoration in the Anthropocene. Mar. Ecol. Prog. Ser. **635**, 203–232. (10.3354/meps13206)

[7] Sweet M, Brown B. 2016 Coral responses to anthropogenic stress in the twenty-first century: an ecophysiological perspective. Oceanogr. Mar. Biol. **54**, 271–314. (10.1201/9781315368597-6)

[8] McLeod IM *et al*. 2022 Coral restoration and adaptation in Australia: the first five years. PLoS One **17**, e0273325. (10.1371/journal.pone.0273325) 36449458 PMC9710771

[9] McLachlan RH, Price JT, Solomon SL, Grottoli AG. 2020 Thirty years of coral heat-stress experiments: a review of methods. Coral Reefs **39**, 885–902. (10.1007/s00338-020-01931-9)

[10] Hughes TP, Tanner JE. 2000 Recruitment failure, life histories, and long-term decline of Caribbean corals. Ecology **81**, 2250–2263. (10.2307/177112)

[11] Baird AH *et al*. 2021 An Indo-Pacific coral spawning database. Sci. Data **8**, 35. (10.1038/s41597-020-00793-8) 33514754 PMC7846567

[12] Miller K, Mundy C. 2003 Rapid settlement in broadcast spawning corals: implications for larval dispersal. Coral Reefs **22**, 99–106. (10.1007/s00338-003-0290-9)

[13] Harrison PL. 2011 Sexual reproduction of scleractinian corals. In Coral reefs: an ecosystem in transition (eds Z Dubinsky, N Stambler), pp. 59–85. Dordrecht, The Netherlands: Springer Netherlands. (10.1007/978-94-007-0114-4_6)

[14] Oliver J, Babcock R. 1992 Aspects of the fertilization ecology of broadcast spawning corals: sperm dilution effects and in situ measurements of fertilization. Biol. Bull. **183**, 409–417. (10.2307/1542017) 29300507

[15] Fogarty ND, Marhaver KL. 2019 Coral spawning, unsynchronized. Science **365**, 987–988. (10.1126/science.aay7457) 31488677

[16] van Woesik R, Lacharmoise F, Köksal S. 2006 Annual cycles of solar insolation predict spawning times of Caribbean corals. Ecol. Lett. **9**, 390–398. (10.1111/j.1461-0248.2006.00886.x) 16623724

[17] Harrison PL, Babcock RC, Bull GD, Oliver JK, Wallace CC, Willis BL. 1984 Mass spawning in tropical reef corals. Science **223**, 1186–1189. (10.1126/science.223.4641.1186) 17742935

[18] Babcock RC, Bull GD, Harrison PL, Heyward AJ, Oliver JK, Wallace CC, Willis BL. 1986 Synchronous spawnings of 105 scleractinian coral species on the Great Barrier Reef. Mar. Biol. **90**, 379–394. (10.1007/BF00428562)

[19] Guest J, Baird A, Goh B, Chou L. 2002 Multispecific, synchronous coral spawning in Singapore. Coral Reefs **21**, 422–423. (10.1007/s00338-002-0263-4)

[20] Cleves PA, Tinoco AI, Bradford J, Perrin D, Bay LK, Pringle JR. 2020 Reduced thermal tolerance in a coral carrying CRISPR-induced mutations in the gene for a heat-shock transcription factor. Proc. Natl Acad. Sci. USA **117**, 28899–28905. (10.1073/pnas.1920779117) 33168726 PMC7682433

[21] Quigley KM, van Oppen MJH. 2022 Predictive models for the selection of thermally tolerant corals based on offspring survival. Nat. Commun. **13**, 1543. (10.1038/s41467-022-28956-8) 35351901 PMC8964693

[22] Quigley KM, Randall CJ, van Oppen MJH, Bay LK. 2020 Assessing the role of historical temperature regime and algal symbionts on the heat tolerance of coral juveniles. Biol. Open **9**, 266255. (10.1242/bio.047316) PMC6994947 31915210

[23] Firkus TJ, Branville C, Neibauer J, Hartleb C, Holmes K, Hauser E, Fischer G. 2024 Induction of out-of-season spawning in an intensively reared walleye (Sander vitreus) broodstock. Aquac. Fish Fish. **4**, e196. (10.1002/aff2.196)

[24] Craggs J, Guest JR, Davis M, Simmons J, Dashti E, Sweet M. 2017 Inducing broadcast coral spawning ex situ: closed system mesocosm design and husbandry protocol. Ecol. Evol. **7**, 11066–11078. (10.1002/ece3.3538) 29299282 PMC5743687

[25] Sakai Y, Yamamoto HH, Maruyama S. 2024 Long-term aquarium records delineate the synchronized spawning strategy of Acropora corals. R. Soc. Open Sci. **11**, 240183. (10.1098/rsos.240183) 39076805 PMC11285485

[26] Martin-Robichaud DJ, Berlinsky DL. 2004 The effects of photothermal manipulation on reproductive development in female haddock Melanogrammus aeglefinus L. Aquac. Res. **35**, 465–472. (10.1111/j.1365-2109.2004.01040.x)

[27] Ramsby BD, Emonnot F, Flores F, Schipper S, Diaz-Pulido G, Abdul Wahab MA, Severati A, Negri AP. 2024 Low light intensity increased survival of coral spat in aquaculture. Coral Reefs **43**, 627–640. (10.1007/s00338-024-02489-6)

[28] Heyward AJ, Negri AP. 1999 Natural inducers for coral larval metamorphosis. Coral Reefs **18**, 273–279. (10.1007/s003380050193)

[29] Webster NS, Smith LD, Heyward AJ, Watts JEM, Webb RI, Blackall LL, Negri AP. 2004 Metamorphosis of a scleractinian coral in response to microbial biofilms. Appl. Environ. Microbiol. **70**, 1213–1221. (10.1128/aem.70.2.1213-1221.2004) 14766608 PMC348907

[30] Abdul Wahab MA, Ferguson S, Snekkevik VK, McCutchan G, Jeong S, Severati A, Randall CJ, Negri AP, Diaz-Pulido G. 2023 Hierarchical settlement behaviours of coral larvae to common coralline algae. Scient. Rep. **13**, 5795. (10.1038/s41598-023-32676-4) PMC10083175 37032381

[31] Hughes DJ, Alexander J, Cobbs G, Kühl M, Cooney C, Pernice M, Varkey D, Voolstra CR, Suggett DJ. 2022 Widespread oxyregulation in tropical corals under hypoxia. Mar. Pollut. Bull. **179**, 113722. (10.1016/j.marpolbul.2022.113722) 35537305

[32] Williamson OM, Allen CE, Williams DE, Johnson MW, Miller MW, Baker AC. 2021 Neighboring colonies influence uptake of thermotolerant endosymbionts in threatened Caribbean coral recruits. Coral Reefs **40**, 867–879. (10.1007/s00338-021-02090-1)

[33] Craggs J, Guest J, Davis M, Sweet M. 2020 Completing the life cycle of a broadcast spawning coral in a closed mesocosm. Invertebr. Reprod. Dev. **64**, 244–247. (10.1080/07924259.2020.1759704)

[34] Sweeney AM, Boch CA, Johnsen S, Morse DE. 2011 Twilight spectral dynamics and the coral reef invertebrate spawning response. J. Exp. Biol. **214**, 770–777. (10.1242/jeb.043406) 21307063

[35] Bauman AG, Baird AH, Cavalcante GH. 2011 Coral reproduction in the world’s warmest reefs: southern Persian Gulf (Dubai, United Arab Emirates). Coral Reefs **30**, 405–413. (10.1007/s00338-010-0711-5)

[36] Okubo N, Motokawa T, Omori M. 2007 When fragmented coral spawn? Effect of size and timing on survivorship and fecundity of fragmentation in Acropora formosa. Mar. Biol. **151**, 353–363. (10.1007/s00227-006-0490-2)

[37] Brady AK, Hilton JD, Vize PD. 2009 Coral spawn timing is a direct response to solar light cycles and is not an entrained circadian response. Coral Reefs **28**, 677–680. (10.1007/s00338-009-0498-4)

[38] Keith SA *et al*. 2016 Coral mass spawning predicted by rapid seasonal rise in ocean temperature. Proc. R. Soc. B **283**, 20160011. (10.1098/rspb.2016.0011) PMC4874704 27170709

[39] Komoto H, Lin CH, Nozawa Y, Satake A. 2023 An external coincidence model for the lunar cycle reveals circadian phase-dependent moonlight effects on coral spawning. J. Biol. Rhythm. **38**, 148–158. (10.1177/07487304221135916) 36461677

[40] Kaniewska P, Alon S, Karako-Lampert S, Hoegh-Guldberg O, Levy O. 2015 Signaling cascades and the importance of moonlight in coral broadcast mass spawning. eLife **4**, e09991. (10.7554/elife.09991) 26668113 PMC4721961

[41] Jokiel PL, Ito RY, Liu PM. 1985 Night irradiance and synchronization of lunar release of planula larvae in the reef coral Pocillopora damicornis. Mar. Biol. **88**, 167–174. (10.1007/BF00397164)

[42] Brady AK, Willis BL, Harder LD, Vize PD. 2016 Lunar phase modulates circadian gene expression cycles in the broadcast spawning coral Acropora millepora. Biol. Bull. **230**, 130–142. (10.1086/BBLv230n2p130) 27132135

[43] Zurl M *et al*. 2022 Two light sensors decode moonlight versus sunlight to adjust a plastic circadian/circalunidian clock to moon phase. Proc. Natl Acad. Sci. USA **119**, e2115725119. (10.1073/pnas.2115725119) 35622889 PMC9295771

[44] Strahl J, Rocker MM, Fabricius KE. 2019 Contrasting responses of the coral Acropora tenuis to moderate and strong light limitation in coastal waters. Mar. Environ. Res. **147**, 80–89. (10.1016/j.marenvres.2019.04.003) 31010596

[45] Anthony KRN, Ridd PV, Orpin AR, Larcombe P, Lough J. 2004 Temporal variation of light availability in coastal benthic habitats: effects of clouds, turbidity, and tides. Limnol. Oceanogr. **49**, 2201–2211. (10.4319/lo.2004.49.6.2201)

[46] Noonan SHC, DiPerna S, Hoogenboom MO, Fabricius KE. 2022 Effects of variable daily light integrals and elevated CO_2_ on the adult and juvenile performance of two Acropora corals. Mar. Biol. **169**, 10. (10.1007/s00227-021-03992-y)

[47] Penland L, Kloulechad J, Idip D, van Woesik R. 2004 Coral spawning in the western Pacific Ocean is related to solar insolation: evidence of multiple spawning events in Palau. Coral Reefs **23**, 133–140. (10.1007/s00338-003-0362-x)

[48] Jones R, Ricardo GF, Negri AP. 2015 Effects of sediments on the reproductive cycle of corals. Mar. Pollut. Bull. **100**, 13–33. (10.1016/j.marpolbul.2015.08.021) 26384866

[49] Mardones ML, Lambert J, Wiedenmann J, Davies TW, Levy O, D’Angelo C. 2023 Artificial light at night (ALAN) disrupts behavioural patterns of reef corals. Mar. Pollut. Bull. **194**, 115365. (10.1016/j.marpolbul.2023.115365) 37579595

[50] Neely KL, Lewis CL, Macaulay KA. 2020 Disparities in spawning times between in situ and ex situ pillar corals. Front. Mar. Sci. **7**, 643. (10.3389/fmars.2020.00643)

[51] Ayalon I *et al*. 2021 Coral gametogenesis collapse under artificial light pollution. Curr. Biol. **31**, 413–419.(10.1016/j.cub.2020.10.039) 33157030

[52] Davies TW, Levy O, Tidau S, de Barros Marangoni LF, Wiedenmann J, D’Angelo C, Smyth T. 2023 Global disruption of coral broadcast spawning associated with artificial light at night. Nat. Commun. **14**, 2511. (10.1038/s41467-023-38070-y) 37188683 PMC10185496

[53] Alderdice R *et al*. 2022 Hypoxia as a physiological cue and pathological stress for coral larvae. Mol. Ecol. **31**, 571–587. (10.1111/mec.16259) 34716959

[54] Edmunds P, Gates R, Gleason D. 2001 The biology of larvae from the reef coral Porites astreoides, and their response to temperature disturbances. Mar. Biol. **139**, 981–989. (10.1007/s002270100634)

[55] Rivest EB, Comeau S, Cornwall CE. 2017 The role of natural variability in shaping the response of coral reef organisms to climate change. Curr. Clim. Chang. Rep. **3**, 271–281. (10.1007/s40641-017-0082-x)

[56] Negri AP, Marshall PA, Heyward AJ. 2007 Differing effects of thermal stress on coral fertilization and early embryogenesis in four Indo Pacific species. Coral Reefs **26**, 759–763. (10.1007/s00338-007-0258-2)

[57] Moreno J. 1993 Physiological mechanisms underlaying reproductive trade-offs. Etologia **3**, 41–56.

[58] Borneman EH. 2000 Aquarium corals: selection, husbandry, and natural history. Neptune City, NJ: TFH Publishing.

[59] Ricardo GF, Jones RJ, Negri AP, Stocker R. 2016 That sinking feeling: suspended sediments can prevent the ascent of coral egg bundles. Scient. Rep. **6**, 21567. (10.1038/srep21567) PMC4761919 26898352

[60] Okubo N, Nakano Y, Mita M. 2020 Lipid composition of gametes in scleractinian reef-building corals: wax-esters generate buoyancy for the gametes. Invertebr. Reprod. Dev. **64**, 291–295. (10.1080/07924259.2020.1815875)

[61] Harii S, Kayanne H, Takigawa H, Hayashibara T, Yamamoto M. 2002 Larval survivorship, competency periods and settlement of two brooding corals, Heliopora coerulea and Pocillopora damicornis. Mar. Biol. **141**, 39–46. (10.1007/s00227-002-0812-y)

[62] Harii S, Nadaoka K, Yamamoto M, Iwao K. 2007 Temporal changes in settlement, lipid content and lipid composition of larvae of the spawning hermatypic coral Acropora tenuis. Mar. Ecol. Prog. Ser. **346**, 89–96. (10.3354/meps07114)

[63] Arai I, Kato M, Heyward A, Ikeda Y, Iizuka T, Maruyama T. 1993 Lipid composition of positively buoyant eggs of reef building corals. Coral Reefs **12**, 71–75. (10.1007/BF00302104)

[64] Leuzinger S, Anthony KRN, Willis BL. 2003 Reproductive energy investment in corals: scaling with module size. Oecologia **136**, 524–531. (10.1007/s00442-003-1305-5) 12802676

[65] Al-Moghrabi S, Allemand D, Couret JM, Jaubert J. 1995 Fatty acids of the scleractinian coral Galaxea fascicularis: effect of light and feeding. J. Comp. Physiol. B **165**, 183–192. (10.1007/BF00260809)

[66] Zhukova NV, Titlyanov E. 2006 Effect of light intensity on the fatty acid composition of dinoflagellates symbiotic with hermatypic corals. Bot. Mar. **49**, 339–346. (10.1515/BOT.2006.041)

[67] Treignier C, Grover R, Ferrier-Pagés C, Tolosa I. 2008 Effect of light and feeding on the fatty acid and sterol composition of zooxanthellae and host tissue isolated from the scleractinian coral Turbinaria reniformis. Limnol. Oceanogr. **53**, 2702–2710. (10.4319/lo.2008.53.6.2702)

[68] Oku H, Yamashiro H, Onaga K, Sakai K, Iwasaki H. 2003 Seasonal changes in the content and composition of lipids in the coral Goniastrea aspera. Coral Reefs **22**, 83–85. (10.1007/s00338-003-0279-4)

[69] Madin JS *et al*. 2016 The Coral Trait Database, a curated database of trait information for coral species from the global oceans. Sci. Data **3**, 160017. (10.1038/sdata.2016.17) 27023900 PMC4810887

[70] Conlan JA, Humphrey CA, Severati A, Parrish CC, Francis DS. 2019 Elucidating an optimal diet for captive Acropora corals. Aquaculture **513**, 734420. (10.1016/j.aquaculture.2019.734420)

[71] Conlan JA, Bay LK, Severati A, Humphrey C, Francis DS. 2018 Comparing the capacity of five different dietary treatments to optimise growth and nutritional composition in two scleractinian corals. PLoS One **13**, e0207956. (10.1371/journal.pone.0207956) 30485343 PMC6261599

[72] Saper J, Høj L, Humphrey C, Bourne DG. 2023 Quantifying capture and ingestion of live feeds across three coral species. Coral Reefs **42**, 931–943. (10.1007/s00338-023-02397-1)

[73] Leuzinger S, Willis BL, Anthony KRN. 2012 Energy allocation in a reef coral under varying resource availability. Mar. Biol. **159**, 177–186. (10.1007/s00227-011-1797-1)

[74] Hennige S, Smith D, Perkins R, Consalvey M, Paterson D, Suggett D. 2008 Photoacclimation, growth and distribution of massive coral species in clear and turbid waters. Mar. Ecol. Prog. Ser. **369**, 77–88. (10.3354/meps07612)

[75] Rapuano H, Shlesinger T, Roth L, Bronstein O, Loya Y. 2023 Coming of age: annual onset of coral reproduction is determined by age rather than size. iScience **26**, 106533. (10.1016/j.isci.2023.106533) 37250314 PMC10214305

[76] Boström-Einarsson L *et al*. 2020 Coral restoration – a systematic review of current methods, successes, failures and future directions. PLoS One **15**, e0226631. (10.1371/journal.pone.0226631) 31999709 PMC6992220

[77] Barton JA, Willis BL, Hutson KS. 2017 Coral propagation: a review of techniques for ornamental trade and reef restoration. Rev. Aquac. **9**, 238–256. (10.1111/raq.12135)

[78] Leal MC, Ferrier‐Pagès C, Petersen D, Osinga R. 2016 Coral aquaculture: applying scientific knowledge to ex situ production. Rev. Aquac. **8**, 136–153. (10.1111/raq.12087)

[79] Omori M. 2005 Success of mass culture of Acropora corals from egg to colony in open water. Coral Reefs **24**, 563. (10.1007/S00338-005-0030-4)

[80] de la Cruz DW, Harrison PL. 2017 Enhanced larval supply and recruitment can replenish reef corals on degraded reefs. Scient. Rep. **7**, 13985. (10.1038/s41598-017-14546-y) PMC5656657 29070842

[81] Suggett DJ, Camp EF, Edmondson J, Boström‐Einarsson L, Ramler V, Lohr K, Patterson JT. 2019 Optimizing return‐on‐effort for coral nursery and outplanting practices to aid restoration of the Great Barrier Reef. Restor. Ecol. **27**, 683–693. (10.1111/rec.12916)

[82] Rinkevich B 2019 The active reef restoration toolbox is a vehicle for coral resilience and adaptation in a changing world. J. Mar. Sci. Eng. **7**, 201. (10.3390/jmse7070201)

[83] Daly J *et al*. 2018 Successful cryopreservation of coral larvae using vitrification and laser warming. Scient. Rep. **8**, 15714. (10.1038/s41598-018-34035-0) PMC6200823 30356142

[84] Hagedorn M, Spindler R, Daly J. 2019 Cryopreservation as a tool for reef restoration: 2019. Adv. Exp. Med. Biol. **1200**, 489–505. (10.1007/978-3-030-23633-5_16) 31471807

[85] Goulet BE, Roda F, Hopkins R. 2017 Hybridization in plants: old ideas, new techniques. Plant Physiol. **173**, 65–78. (10.1104/pp.16.01340) 27895205 PMC5210733

[86] Lamb AM *et al*. 2024 Fertile hybrids could aid coral adaptation. Ecol. Evol. **14**, e70570. (10.1002/ece3.70570) 39568767 PMC11578633

[87] Koukoumaftsis LP, Salmon M, Everson G, Hughes DJ, Varkey D, Heyward AJ, Severati A, Humphrey C. 2025 Data from: Controlled out-of-season spawning of reef-forming corals using offset environmental cues. Dryad Digital Repository. (10.5061/dryad.v15dv4281)40957569

[88] Koukoumaftsis LP, Salmon M, Everson G, Hughes DJ, Varkey D, Heyward A *et al*. 2025 Supplementary material from: Controlled out-of-season spawning of reef-forming corals using offset environmental cues. Figshare. (10.6084/m9.figshare.c.7988973)40957569

